# Volume‐Stable Collagen Matrix With or Without Platelet‐Rich Fibrin Impregnation for Multiple Gingival Recessions: A Randomized Clinical Trial

**DOI:** 10.1111/jre.70127

**Published:** 2026-06-25

**Authors:** Gabriel Leonardo Magrin, Roberta Michels, Ana Clara Kuerten Gil, Kelly Batista Maier, Bruna Mueller, Reinhard Gruber, Cesar Augusto Magalhães Benfatti

**Affiliations:** ^1^ Centre for Education and Research on Dental Implants (CEPID), Department of Dentistry Federal University of Santa Catarina (UFSC) Florianópolis Brazil; ^2^ Department of Periodontology University of Bern Bern Switzerland; ^3^ Department of Oral Biology Medical University of Vienna Vienna Austria

**Keywords:** collagen matrix, gingival recession, periodontology, platelet‐rich fibrin, randomized controlled trial, root coverage

## Abstract

**Aim:**

This split‐mouth randomized clinical trial aimed to assess whether incorporating platelet‐rich fibrin (PRF) into a volume‐stable collagen matrix results in superior reduction of recession depth after 6 months of healing in the treatment of multiple gingival recessions using the coronally advanced flap.

**Methods:**

Patients with bilateral maxillary RT1 recessions at non‐molar sites were recruited. Each side received either a PRF‐impregnated matrix or a non‐impregnated matrix. Clinical parameters, including recession depth, keratinized tissue width, bleeding on probing, probing depth, clinical attachment level, and gingival thickness, were measured at baseline and 6 months, as well as the Root Coverage Esthetic Score (RES) and patient‐reported outcome measures (PROMs). The percentage of complete root coverage was calculated 6 months after treatment.

**Results:**

Twenty‐five patients were enrolled. However, one patient was excluded due to incomplete follow‐up. Both groups significantly improved over time, but no intergroup differences were observed for any of the clinical parameters (*p* > 0.05). No statistically significant differences were found between test and control groups in evaluations of RES and PROMs, showing similar postoperative experiences of patients.

**Conclusion:**

Within the limitations of this study, adding PRF to a volume‐stable collagen matrix did not result in superior recession depth reduction or improved clinical, esthetic, or patient‐reported outcomes after 6 months.

**Trial Registration:**

Brazilian Register of Clinical Trials (ensaiosclinicos.gov.br) identifier: RBR‐3hqrvrr (20/10/2021)

## Clinical Relevance Box

**Background:**

Soft tissue augmentation using collagen matrices combined with the coronally advanced flap (CAF) technique has emerged as an alternative to connective tissue grafts for treating multiple gingival recessions. Platelet‐rich fibrin (PRF) has been proposed to enhance soft tissue regeneration through the release of growth factors that promote angiogenesis and epithelial differentiation. However, its clinical benefits when combined with collagen matrices remain uncertain.

**Added Value of This Study:**

This is the first clinical trial that evaluated the effect of PRF impregnation of a volume‐stable collagen matrix on clinical, esthetic, and patient‐reported outcomes in multiple gingival recessions. Both treatment groups, CAF with or without PRF, demonstrated significant improvements in recession depth, attachment level, and esthetics after 6 months, but no statistically significant intergroup differences were found.

**Clinical Implications:**

The addition of PRF did not enhance root coverage outcomes compared with collagen matrix alone, indicating limited clinical value in this setting.

## Graphical abstract

This split‐mouth randomized clinical trial showed that platelet‐rich fibrin impregnation of a volume‐stable collagen matrix did not improve clinical, esthetic, or patient‐reported outcomes compared with collagen matrix alone for multiple gingival recession treatment after 6 months.

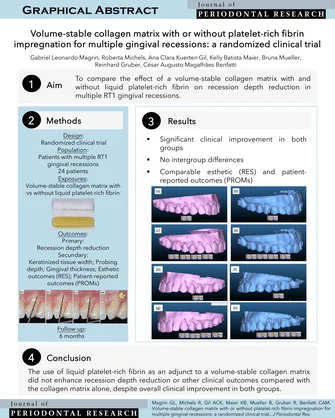

## 1 Introduction

Gingival recessions are a highly prevalent condition, affecting approximately 81% of the general population [1], and consist of the apical migration of the gingiva relative to the cementoenamel junction associated with the loss of supporting periodontium and root exposure to the oral environment [2]. Several aspects of gingival recession make it clinically significant, such as its potential influence on dentin hypersensitivity, predisposition to root caries, and possible esthetic repercussions [3, 4, 5]. A large number of techniques are available for root coverage supported by substantial scientific literature [6, 7, 8, 9]. A long‐term effectiveness of root coverage for multiple recessions have been reported with coronally advanced flap technique associated to connective tissue grafting [4, 9, 10, 11, 12]. However, the use of autogenous soft tissue presents limitations related to graft size, including restrictions in height, length, and thickness of the harvested tissue, patient‐specific anatomical factors, proximity to vital structures in the palatal donor area, and risk of postoperative complications, such as hemorrhage or even necrosis at the donor site [13, 14]. In this context, xenogeneic collagen matrices emerged as an alternative to connective tissue grafts.

Substitute materials require long‐term volumetric stability and favorable biological performance to promote remodeling and mimic the autogenous graft [15]. Three‐dimensional (3D) stable collagen matrices have been developed for soft tissue graft replacement, showing positive results in terms of fibroblast growth, integration into adjacent tissues, and stimulation of angiogenesis [16], demonstrating no inferiority compared to connective tissue grafts and exhibiting longitudinal volumetric stability [13, 14, 15, 17]. Although their potential for tissue formation, concerns remain regarding the biological limitations of collagen matrices compared to connective tissue, particularly due to its acellular nature, which may lead to unpredictable clinical outcomes such as the failure to generate keratinized mucosa [13, 18].

Platelet‐rich fibrin (PRF) has been widely studied in Periodontology for its ability to enhance tissue formation, serving as a matrix rich in growth factors, such as transforming growth factor (TGF)‐β, that supports cell migration and differentiation [19, 20]. Liquid PRF can be obtained by modifying the blood collection tube, as well as the centrifugation time and speed [21]. This platelet concentrate may enhance vascularization, which facilitates the sustained release of growth factors, thereby promoting further cellular growth factor secretion [22, 23, 24]. Given these biological effects, integrating PRF into a collagen matrix becomes a biologically plausible strategy to enhance cellular differentiation and epithelial maturation for gingival tissue formation [25]. Previous clinical reports have explored the functionalization of volume‐stable collagen matrices with liquid PRF for the treatment of gingival recessions. This approach was first documented for the treatment of a single gingival recession, reporting promising results for a 6‐month follow‐up clinical case [26]. A subsequent case report from our group presented a similar method for the treatment of multiple gingival recessions, with a mean root coverage of 83% at 12‐month follow‐up, and stable outcomes maintained up to 30 months [27]. Thus, these findings suggest that PRF impregnation into a collagen matrix may serve as a potential alternative to autogenous grafts in the treatment of multiple gingival recessions.

Considering the capacity of PRF to enhance cellular colonization and support tissue maturation, our hypothesis was that its addition to a volume‐stable collagen matrix would enable undifferentiated mesenchymal cells to promote the development of a thick, keratinized epithelial phenotype, potentially enhancing the long‐term stability of root coverage outcomes. The primary endpoint of this study was to evaluate whether the incorporation of PRF into a collagen matrix would enhance recession depth reduction after 6 months of healing in multiple gingival recession treatment using the coronally advanced flap technique compared with the same collagen matrix without PRF. Secondary endpoints included the assessment of clinical parameters influencing root coverage outcomes, such as keratinized tissue formation, increase in gingival thickness, and changes in gingival phenotype, among others, along with esthetic results and patient‐reported experiences from the procedure. The null hypothesis was that the incorporation of PRF into a volume‐stable collagen matrix would not result in additional recession depth reduction as compared to the collagen matrix without PRF.

## 2 Methods

### 2.1 Ethical Aspects

This study received ethical approval from the ethics committee of the Federal University of Santa Catarina (UFSC; protocol number 4.735.476). All participants signed an informed consent form before inclusion in the study. This investigation complies with the Declaration of Helsinki as revised in 2013, and the ICH Good Clinical Practice Guidelines. The research protocol was registered in a clinical trial registry before initiation (Brazilian Register of Clinical Trials; register identifier RBR‐3hqrvrr). The present study was reported following the CONSORT guidelines [28], and adheres to the Principled Framework for Mendelian Randomization in Oral Health Research [29].

### 2.2 Participants

For this prospective, superiority framework, split‐mouth randomized controlled clinical trial, with patient blinding and blinded data analysis. Clinical outcome measurements were performed by the primary surgeon (G.L.M.), who was therefore not blinded to treatment allocation. Patients with multiple gingival recessions were selected at the dental clinic of UFSC between October 2021 and October 2023. The inclusion criteria considered bilateral marginal deficiencies in multiple and homologous sites of the maxillary esthetic zone (between teeth 15 and 25) without interproximal involvement (Recession Type 1—RT1) [30]. A minimum of three adjacent teeth on each side of the maxilla had to present marginal recession, with at least one of them having a recession depth ≥ 3 mm. Patients with gingival recessions in molars were included; however, molar sites were not considered for data analysis. When present, non‐carious cervical lesions (NCCLs) were restored prior to surgery by a restorative dentistry specialist (B.M.). The cementoenamel junction (CEJ) was reconstructed using composite resin restorations to recreate the natural morphology and establish an appropriate emergence profile while avoiding excessive convexity. When the homologous tooth exhibited an intact CEJ, it served as the primary reference. In cases where both the treated and homologous teeth presented CEJ abrasion, the reference was established through morphological and proportional analysis based on crown dimensions and the estimation of the expected root coverage. Baseline keratinized tissue width was not used as an inclusion criterion. However, recessions with < 1 mm keratinized tissue band apical to the defect were excluded. Patients must be at least 18 years old, present a good biofilm control (plaque index < 25%), and have no baseline conditions that could affect tissue healing.

The exclusion criteria considered uncontrolled local or systemic conditions, such as diabetes, pregnancy, and periodontitis. Smokers of > 10 cigarettes/day were also removed from the study. Additionally, participants were excluded if they had a history of mucogingival or periodontal surgery, dental implants, prosthetic crowns on teeth at the surgical sites, or gingival recessions with < 1 mm of keratinized tissue apical to the recession areas.

### 2.3 Randomization

A randomization sequence was generated on an online platform (randomization.com) by an investigator not involved in the clinical stages of this research (R.M.). This sequence assigned the study treatments (test and control) to the surgical sites of each patient (right and left), and was distributed in sealed, opaque, and numbered black envelopes. During the surgical procedure, after surgical site preparation and immediately before the placement of the collagen matrices, the corresponding envelope was opened and the treatment allocation for each side was revealed.

### 2.4 Sample Size Calculation

The sample size calculation was performed using the open‐access software G* Power 3.1.9.2 (University of Düsseldorf, Germany) based on recession depth reduction (primary outcome) and considering a superiority trial design. Given a recession reduction of 3.0 ± 0.5 mm associated with collagen matrices after 6 months [31], the sample size calculation showed that 22 patients were needed for a split‐mouth study design (*α* = 5%, power = 80%, standardized effect size = 0.63). Adding a 10% dropout rate, a total of 25 patients composed the final sample for this clinical trial.

### 2.5 Preoperative Protocol and Clinical Measurements

Before surgery, in the operating room, a nurse collected venous blood from the patients from the median cubital vein into plastic tubes, which were immediately placed in a centrifuge (Intra‐Lock L‐PRF Intra Spin, Birmingham, AL, USA) at a speed of 2700 rpm, 400 g of Relative Centrifugal Force, for 3 min. The tubes were then removed from the centrifuge, and the liquid PRF (fibrinogen) was aspirated with a pipette. Meanwhile, the surgeon prepared the volume‐stable collagen matrix (20 × 40 × 6 mm Fibro‐Gide, Geistlich Pharma, Wolhusen, Switzerland) by cutting it in half vertically (approximately 10 mm) and trimming the thickness to about 3 mm, obtaining two parts with the same dimensions (Figure 1a–c). This trimming approach followed a clinical recommendation from a previous study [31], and intended to enhance flap adaptation, reduce tension, and allow complete biomaterial coverage, minimizing the risk of early wound dehiscence. One part of the hemisected matrix was impregnated with the PRF from the syringe, while the other was hydrated in saline solution for 20 min (Figure 1d–g).

**FIGURE 1:**
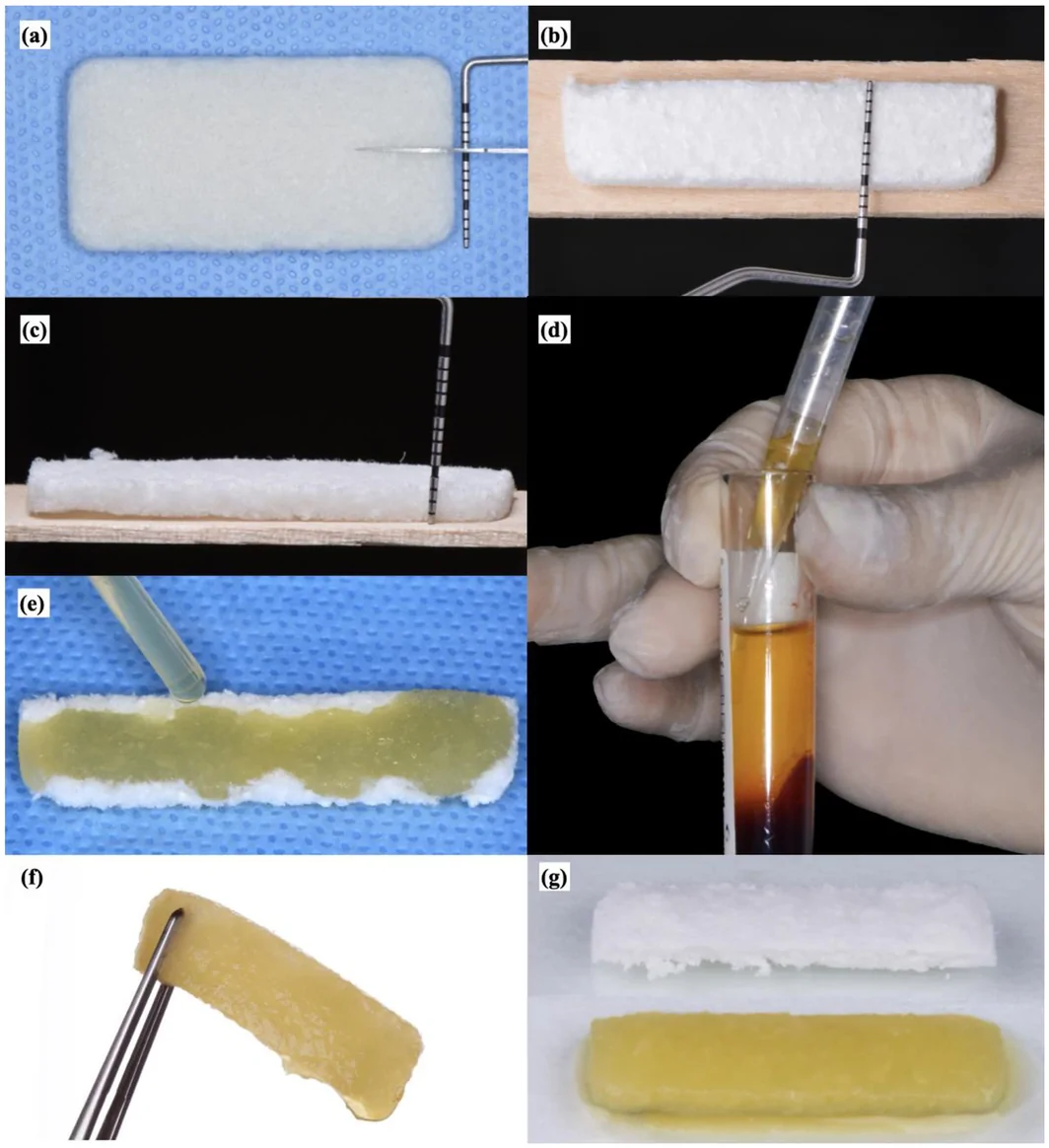
Preparation of the volume‐stable collagen matrix (VSCM): (a) Original matrix dimensions (20 × 40 × 6 mm); (b, c) Matrix trimmed to final dimensions (10 × 40 × 3 mm); (d) Liquid PRF aspiration; (e, f) PRF impregnation into one portion of the matrix; (g) Prepared matrix, one portion (upper in the image) resting dry for saline solution immersion (control group) and another portion (lower in the image) after PRF impregnation (test group).

Before local anesthesia, standardized photographs of the surgical sites were taken (Nikon D3200, Nikon AF‐S VR Micro‐NIKKOR 105 mm f/2.8G IF‐ED lens, Sigma EM −140 DG ring flash; Tokyo, Japan) using lip retractors and a black background to allow a clear view of the teeth and surrounding soft tissues, as well as the mucogingival junction. A maxillary full‐arch impression was obtained for each patient and cast in Type V dental stone to subsequent digital model creation for the bilateral assessment of multiple gingival recessions.

For clinical measurements, each participant contributed with two teeth, one on each side of the maxilla, those with the deepest recessions (Figure S1). Adjacent recessions in molar sites were not included for clinical measurements but were treated within the same surgical intervention. The following clinical measurements were performed by an experienced periodontist (G.L.M.):

- Recession depth (RD) from gingival margin to cementoenamel junction at the mid‐buccal site;
- Keratinized tissue width (KTW) from gingival margin to mucogingival junction at the mid‐buccal site:
- Bleeding on probing (BOP) at the mid‐buccal site;
- Probing depth (PD) at the mid‐buccal site:
- Clinical attachment level (CAL) at the mid‐buccal site;
- Gingival thickness (GT) was assessed using an injection needle positioned at the center of a silicone stop measuring 3.0 mm in diameter. The alignment of the silicone stop border with the gingival margin resulted in a penetration point located approximately 1.5 mm apical to the gingival margin. The needle was then introduced perpendicularly through the soft tissue until touching the root surface underneath. After needle insertion, flowable composite resin was applied over the silicone stop to prevent displacement.
- Gingival phenotype (GP) estimated by the transparency of the periodontal probe [5]. When the periodontal probe was visible through the gingival tissue, the phenotype was categorized as thin. When the probe was not visible through the gingiva, the phenotype was classified as thick.

Except for GT, all measurements were performed using a 15‐mm periodontal probe (University of North Carolina Periodontal Probe, UNC‐PCP 15, Helmut Zepf, Seitingen‐Oberflacht, Germany).

### 2.6 Surgical Procedure

The surgical procedure comprised a coronally advanced flap with a volume‐stable collagen matrix (VSCM) impregnated with PRF (test) on one side of the maxilla and a coronally advanced flap with the same collagen matrix without PRF (control) on the other side. Two surgeons experienced in periodontal surgery were responsible for the procedures (G.L.M. = 22; C.A.M.B. = 3). Prior to study initiation, both surgeons aligned the surgical protocol to standardize flap design, dissection sequence, root surface preparation, matrix handling, and suturing technique. All procedures were performed according to a predefined protocol to ensure inter‐operator consistency. Given the single‐center design and the limited number of operators, no formal calibration exercise was conducted.

The flap design followed established techniques for the treatment of multiple adjacent recessions [32]. After local anesthesia (2% mepivacaine with 1:100000 epinephrine), intrasulcular incisions with no papilla involvement were made, including at least one mesial and one distal tooth to the gingival recession sites, without vertical incisions. Oblique incisions were made in the interproximal soft tissues, and a partial‐thickness flap was elevated (Figure 2a,i). Then, a full‐thickness flap was raised to the mucogingival junction (Figure 2b,j). The soft tissue was then mobilized with horizontal incisions beyond the mucogingival junction to relieve muscle tension and allow coronal advancement of the gingival margin (Figure 2c,k). The root surfaces were carefully instrumented, and the interproximal papilla was de‐epithelialized (Figure 2d,e,l,m). Allocation of surgical sites to the study groups was then performed in accordance with the randomization protocol.

**FIGURE 2:**
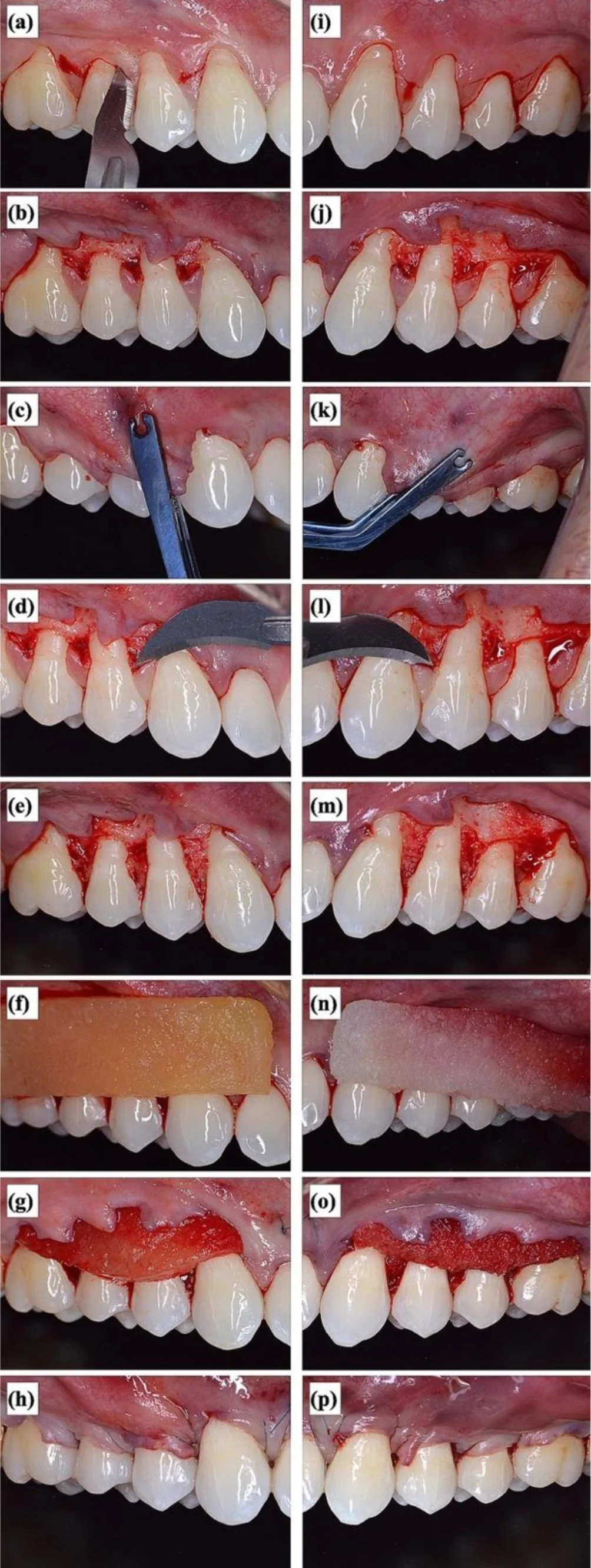
Surgical procedure in test (left column) and control (right column) groups: (a, i) Intrasulcular and oblique incisions; (b, j) Partial and then full‐thickness flap elevated to the mucogingival junction; (c, k) Horizontal incisions beyond the mucogingival junction to relieve muscle tension and allow coronal advancement of the gingival margin; (d, e, l, m) Papilla de‐epithelialization; (f) Collagen matrices with PRF and (n) without positioned at the cementoenamel junction and (g, o) sutured mesially and distally; (h, p) Flap coronally advanced and stabilized with sling sutures, fully covering the matrix.

Volume‐stable collagen matrices were applied at the coronal level of the cementoenamel junction (natural or previously restored with composite resin) (Figure 2f,n). The collagen matrices were sutured to the surgical bed at their mesial and distal ends with simple interrupted sutures using a non‐absorbable 5–0 blue nylon monofilament (Techsuture, São Paulo, SP, Brazil) (Figure 2g,o). The surgical flap was then passively positioned coronally to the cementoenamel junction of involved teeth, and sling sutures involving the surgical papilla were used to stabilize the flap (5–0 blue nylon monofilament, Techsuture). Collagen matrices were entirely covered by the flap, with no exposed areas (Figure 2h,p). Patients were unaware of which groups were allocated to each side of the maxilla.

### 2.7 Postoperative Protocol, PROMs and Follow‐Up

Immediately after surgery, patients were instructed on postoperative care. The medication protocol included amoxicillin 500 mg every 8 h for 7 days, ibuprofen 600 mg every 8 h for 3 days, and 0.12% chlorhexidine digluconate every 12 h for 10 days, starting on the third postoperative day. Chlorhexidine rinsing was initiated on postoperative Day 3 to avoid disruption of clot stabilization, flap adhesion, and early cellular migration during the critical initial healing phase [33]. In vivo evidence indicates that the first 24 h represent a biologically active period in gingival wound healing, during which cellular proliferation and apoptotic pathways are established [34]. Therefore, postponing chlorhexidine initiation aimed to minimize possible modulation of early healing dynamics while still ensuring adequate chemical plaque control thereafter. Although routine antibiotic prescription following coronally advanced flap procedures is not supported by strong evidence, this regimen was adopted in the present study to maintain consistency with previous graft‐based clinical trials [15, 35], and to reduce the risk of early postoperative complications.

One week after surgery, patients were invited to return for follow‐up. At this visit, patient‐reported outcome measures (PROMs) were collected using a structured questionnaire, which consisted of seven items: (Q1) intraoperative discomfort; (Q2) intraoperative pain; (Q3) postoperative pain; (Q4) postoperative bleeding; (Q5) swelling; (Q6) facial bruising, and (Q7) patient's overall perception of postoperative discomfort [36]. For questions Q1‐Q6, patients recorded the intensity of each symptom separately for the right and left sides of the maxilla using a 100‐mm visual analogue scale (VAS), where 0 indicated “no symptom” and 100 indicated “maximum intensity.” This side‐specific approach ensured that patients, who remained blinded to the treatment allocation, could provide an independent evaluation of each surgical site. Item Q7 asked patients to indicate which side was perceived as less uncomfortable overall. Values < 50% represented the test side and values > 50% the control side. PROMs were collected by an examiner (A.C.K.G. or K.B.M.) not involved in the surgical procedures to minimize interviewer bias. Patients returned 2 weeks after surgery for suture removal.

Patients were scheduled for follow‐up and treatment outcome assessment 6 months after root coverage. Photographs of the surgical sites were taken with the same settings of pre‐surgical phase. An additional maxillary full‐arch impression was obtained at the 6‐month follow‐up visit and another stone model was produced. The same clinical measurements assessed preoperatively were collected, adding the percentage of root coverage (%RC), which represents the proportion of the root covered by soft tissue, calculated from the gingival margin at baseline to 6 months post‐surgery, considering the cementoenamel junction as the 100% reference point, and the percentage of complete root coverage (%CRC), which was the proportion of teeth that reach full coverage of the exposed root.

### 2.8 Esthetic Assessment

Photos taken at baseline and 6 months after root coverage were exported to a single computer and used for calibration and analysis through the Root Coverage Esthetic Score (RES). After training with an experienced periodontist (G.L.M.), two calibrated examiners (K.B.M. and B.M.) independently assessed the images according to the predefined RES criteria. Calibration was performed in a single session after completion of the 6‐month follow‐up, using standardized baseline and 6‐month photographs from 24 patients. One patient was later excluded from the final analysis due to absence at the 6‐month evaluation. Inter‐examiner reliability for the total RES score was assessed. To assess intra‐examiner reliability, each examiner repeated the RES evaluation after a 2‐week interval using the same images. In cases of disagreements, a third party (G.L.M.) was consulted to determine the final score.

The RES was based on five parameters: gingival margin (GM), marginal tissue contour (MTC), soft tissue texture (STT), mucogingival junction (MGJ) alignment, and gingival color (GC). Zero, 3, or 6 points were used for the evaluation of the position of GM, whereas a score of 0 or 1 point was used for each of the other variables. Zero points were assigned if the final position of GM was equal to or apical to the previous recession depth (failure of root coverage procedure), irrespective of color, the presence of a scar, MTC, or MGJ. Zero points were also assigned when a partial or total loss of interproximal papilla (black triangle) occurred following the treatment [37].

### 2.9 Root Coverage Assessment in Digital Models

Stone casts from each patient were digitized using an Identica Hybrid 3D scanner (Medit, Seoul, South Korea). Root coverage of the treated teeth was analyzed using the open‐source 3D point cloud and mesh processing software CloudCompare (version 2.14 alpha; CloudCompare, EDF R&D/Telecom, Paris, France). The reference tooth as well as the group of teeth involved in the surgical procedure on each side of the maxilla (test and control) were included in the analysis for each patient. Measurements were performed using the “Point Picking” tool by drawing a straight line from the cementoenamel junction to the gingival margin at the central portion of the tooth. One examiner (A.C.K.G.) obtained all measurements after training with another researcher (G.L.M.), but no intra‐examiner reliability assessment was performed.

Measurements were obtained at baseline and at follow‐up digital models, and the difference between these time points was considered the linear root coverage expressed in millimeters. Considering the multiple teeth involvement, the mean value of the individual measurements was calculated for each side of the maxilla, excluding the molars (Figure 3).

**FIGURE 3:**
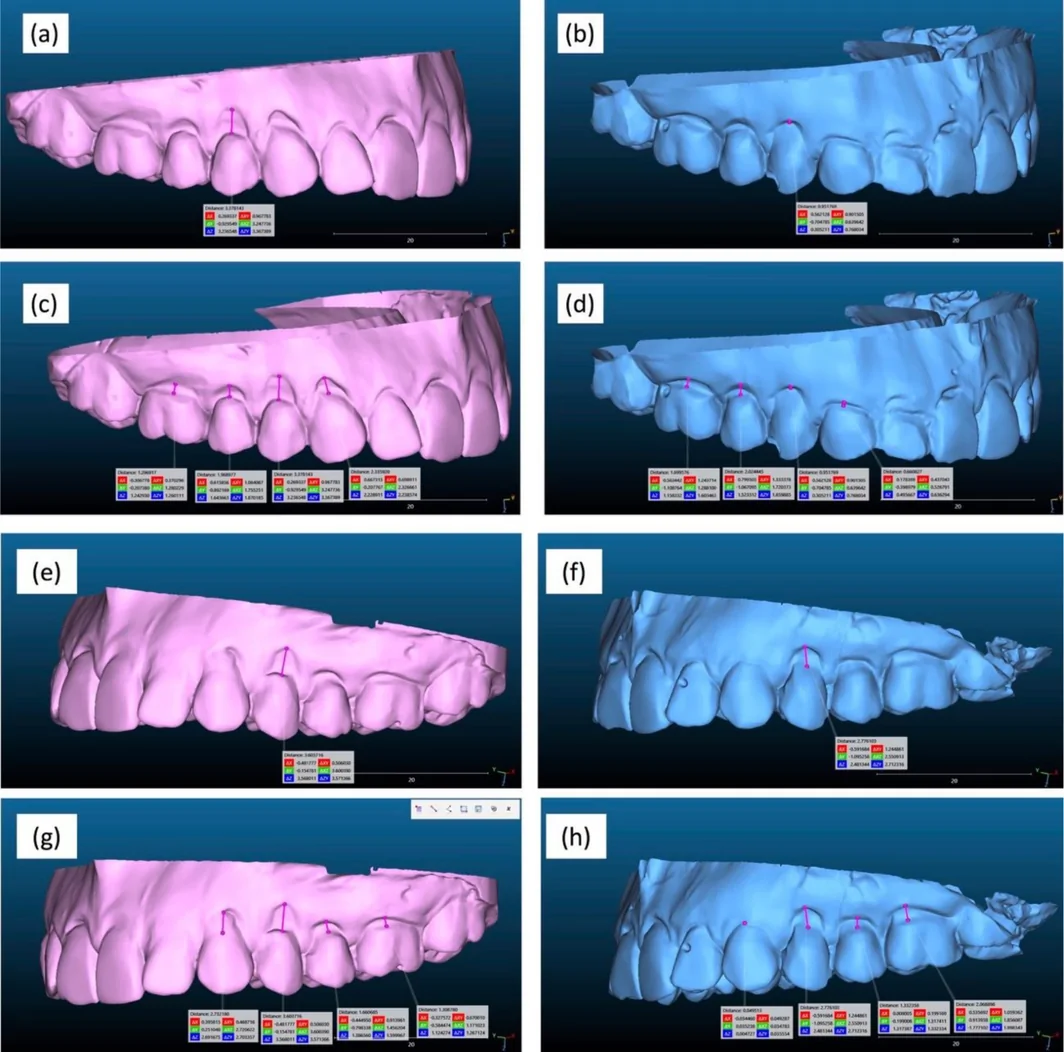
Analysis of digital models performed in CloudCompare. (a–d) right side (test group). (e–h) left side (control group). Measurements are illustrated for single reference teeth (a, b, e, f) and for multiple recession teeth (c, d, g, h). Pink models represent baseline measurements, while blue models represent follow‐up measurements, with linear distances indicated by reference markers, in mm. Molars were measured but were not considered in this study.

### 2.10 Data Analysis

Collected data were summarized descriptively to illustrate the distribution of outcomes across patients and experimental groups. Individual root coverage percentages were calculated for each treated tooth. Statistical analyses were conducted using GraphPad Prism (Version 10.4.1, GraphPad Software, San Diego, CA, USA). The Wilcoxon test was used to perform data analysis. Data were calculated along with its 95% confidence interval (95% CI). Inter‐ and intra‐examiner reliability were assessed using the intraclass correlation coefficient (ICC) for the secondary outcome of RES. Considering the continuous nature of the total score, a two‐way random‐effects model with absolute agreement was applied for ICC. Due to the split‐mouth design, each patient contributed two independent observations, resulting in a total of 48 paired assessments. All analyses assumed a significance level of 5% (*p* = 0.05).

## 3 Results

### 3.1 Patient‐Related Aspects

Seventy‐six individuals were assessed for inclusion. However, 51 patients could not be included for various reasons, such as presence of dental implant in the area of interest, absence of bilateral recessions, interproximal bone loss, periodontitis, among others. Twenty‐five patients underwent surgical intervention. One patient dropped out at the 6‐month follow‐up due to personal reasons (moving abroad). Therefore, 24 participants (female = 11, male = 13) with an age range from 22 to 53 years old (mean age = 31.87 years) completed the 6‐month follow‐up evaluation (last follow‐up in November 2024). Each patient contributed one reference tooth per side (test and control), corresponding to the deepest recession site. More details in Figure 4, Table 1.

**FIGURE 4:**
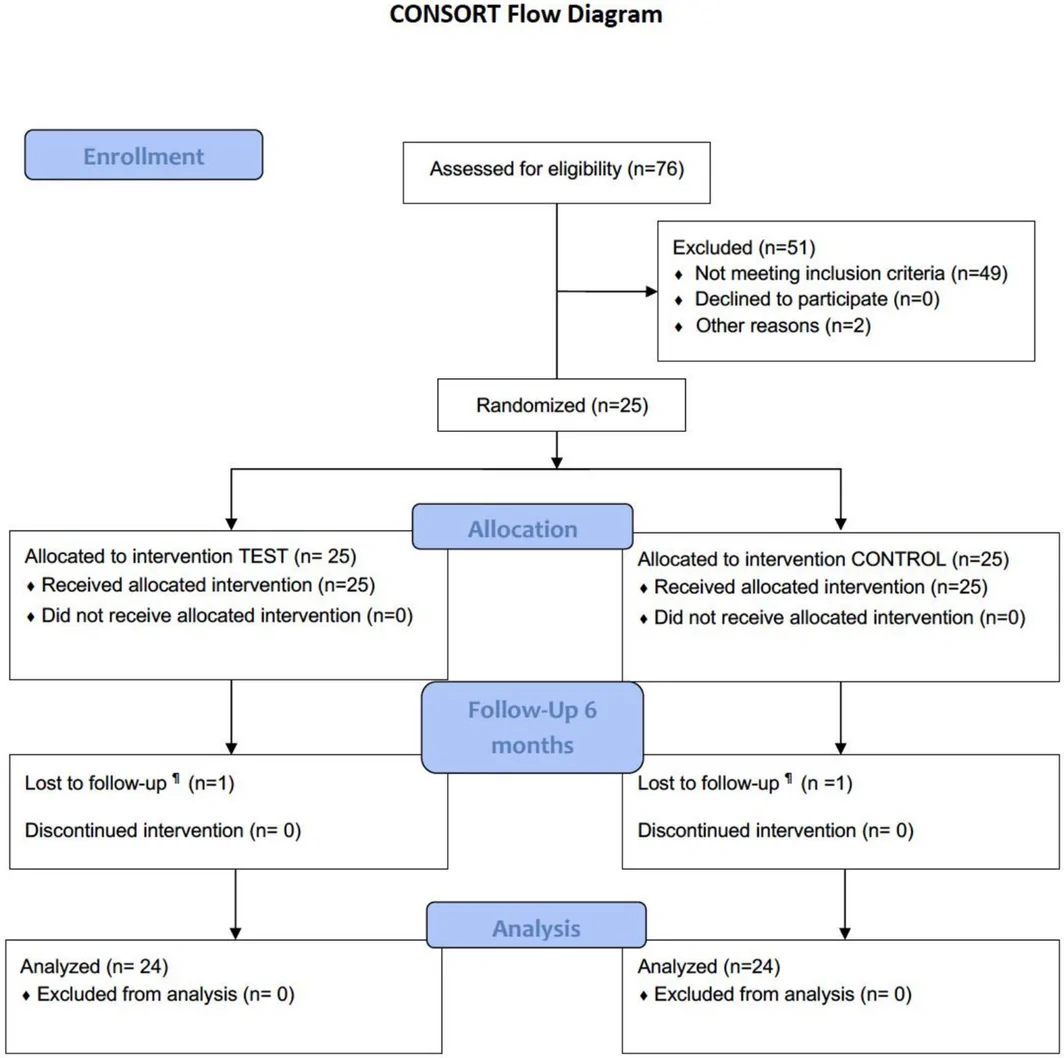
CONSORT flowchart. ^¶^The patient discontinued during the follow‐up.

**TABLE 1:** Patient characteristics and root coverage percentage of reference teeth.

Patient	Sex	Age	Reference tooth		%RC	
			Test	Control	Test	Control
1	M	37	14	24	33%	33%
2	F	25	23	13	0%	0%
3	M	48	24	14	50%	50%
4	M	39	13	23	0%	42.5%
5	M	32	14	24	0%	−14.3%
6	F	23	25	13	42.9%	100%
7	F	27	24	14	33.3%	42.9%
8	M	26	14	24	50%	50%
9	M	34	23	14	50%	50%
10	F	41	23	14	40%	50%
11	M	27	13	24	100%	100%
12	M	23	14	24	100%	100%
13	M	34	24	14	66.6%	50%
14	F	27	14	23	100%	100%
15	M	28	13	24	85.7%	100%
16	M	35	14	24	75%	100%
17	M	48	23	13	33.3%	57.1%
18	F	44	14	24	33.3%	100%
19	F	53	24	13	66.7%	75%
20	F	32	24	14	66.7%	66.7%
21	F	44	23	15	80%	25%
22	F	36	14	24	50%	60%
23	F	35	13	23	66.7%	50%
24	M	25	14	25	66.7%	33.6%

*Note:* (M) Male, (F) Female; %RC: Percentage of root coverage in the reference tooth.

### 3.2 Clinical Parameters

The primary outcome of this trial was RD reduction from baseline to 6 months. Both groups showed significant intragroup RD reduction. In the test group, mean RD decreased from 3.56 ± 0.81 mm at baseline to 1.85 ± 1.35 mm at 6 months (mean reduction: −1.70 ± 1.25 mm; *p* < 0.001). In the control group, RD decreased from 3.52 ± 0.96 to 1.50 ± 1.17 mm (mean reduction: −2.02 ± 1.41 mm; *p* < 0.001). No statistically significant difference was identified between groups regarding RD reduction at 6 months (*p* = 0.408).

Paired analysis within the test group (Figure S2) revealed statistically significant improvements in RD and CAL from baseline to the 6‐month follow‐up. Increases in PD and GT were also observed. No significant differences were found for KTW or BOP, while GP improved, with most sites transitioning from thin to thick phenotype. In the control group (Figure S3), RD, CAL, and GT showed improvements. A reduction in KTW was observed, while PD and BOP showed no significant differences from baseline. As with the test group, GP exhibited a favorable shift toward a thick phenotype by the 6‐month evaluation. No statistically significant differences were observed between test and control groups for any of the clinical parameters examined at the 6‐month follow‐up (Figure S4), including RD, KTW, BOP, PD, CAL, GT, %RC, or %CRC. Both groups demonstrated similar clinical improvements, regardless of the approach. More details in Table 2.

**TABLE 2:** Mean value, standard deviation, median, percentiles, and *p*‐value for test and control groups at baseline and 6 months obtained from clinical measurements.

Clinical parameters		Test	Median (Q1‐Q3)	p value (BL vs 6 months)	Control	Median (Q1‐Q3)	p value (BL vs 6 months)	p value (Test vs Control 6 months)
RD (mm)	Baseline	3.56 (0.81)	3.00 (3.00–4.00)	< 0.001	3.52 (0.96)	3.00 (3.00–4.00)	< 0.001	0.408
	6 months	1.85 (1.35)	1.50 (1.00–2.75)		1.50 (1.17)	2.00 (0.00–2.00)		
	Difference	−1.70 (1.25)			−2.02 (1.41)			
KTW (mm)	Baseline	2.62 (1.50)	2.00 (1.12–4.00)	0.163	2.25 (1.23)	2.00 (1.12–3.00)	0.046	0.247
	6 months	2.12 (1.30)	2.00 (1.00–3.00)		1.64 (1.06)	2.00 (1.00–2.87)		
	Difference	−0.50 (1.62)			−0.60 (1.32)			
BOP (%)	Baseline	16.66		> 0.999	12.5		> 0.453	0.688
	6 months	16.66			25			
PD (mm)	Baseline	1.62 (0.71)	1.50 (1.00–2.00)	0.016	1.79 (0.58)	2.00 (1.00–2.00)	> 0.999	0.826
	6 months	1.91 (0.77)	2.00 (1.00–2.75)		1.83 (0.56)	2.00 (1.25–2.00)		
	Difference	+0.29 (0.46)			+0.04 (0.35)			
CAL (mm)	Baseline	4.81 (0.74)	5.00 (4.00–5.00)	< 0.001	5.12 (0.96)	5.00 (4.12–6.00)	< 0.001	0.847
	6 months	3.58 (1.13)	3.50 (1.00–4.00)		3.64 (1.12)	3.75 (3.00–4.75)		
	Difference	−1.22 (1.25)			−1.47 (1.37)			
GT (mm)	Baseline	0.86 (0.41)	1.00 (0.50–1.04)	0.005	0.89 (0.350)	0.80 (0.70–1.00)	< 0.001	0.268
	6 months	1.15 (0.36)	1.05 (1.00–1.50)		1.24 (0.43)	1.20 (0.92–1.50)		
	Difference	+0.27 (0.40)			+0.35 (0.35)			
GP (%)	Baseline	79.16 (thin)/20.83 (thick)		< 0.001	83.33 (thin)/16.66 (thick)		< 0.001	0.125
	6 months	16.66 (thin)/83.33 (thick)			33.33 (thin)/66.66 (thick)			
RC (%)	6 months	53.76 (29.58)			59.84 (31.29)			0.289
CRC (%)	6 months	12.50 (33.08)			29.17 (45.42)			0.164

*Note:* Wilcoxon test was applied for statistical analysis.

Abbreviations: −, decreased; +, increased; *BL*, baseline; BOP, bleeding on probing; CAL, clinical attachment level; CRC, complete root coverage, GP, gingival phenotype; GT, gingival thickness; KTW, keratinized tissue width; PD, probing depth; Q1, 25% percentile; Q3, 75% percentile; RC, root coverage; RD, recession depth.

### 3.3 Questionnaire‐Related Data

In the PROMs evaluation, each symptom was analyzed separately for the right and left sides. Mean VAS scores for the test and control groups, respectively, were as follows: intraoperative discomfort (4.13 ± 2.47 vs. 3.50 ± 2.50), intraoperative pain (2.57 ± 2.42 vs. 3.00 ± 2.79), postoperative pain (2.52 ± 2.56 vs. 3.00 ± 3.17), postoperative bleeding (1.60 ± 2.27 vs. 1.79 ± 2.17), swelling (3.68 ± 2.79 vs. 4.92 ± 3.12), and facial bruising (1.66 ± 2.39 vs. 2.18 ± 3.11). No statistically significant differences were detected between groups for any parameter (*p* > 0.05). Regarding overall postoperative perception (Q7), 12 patients reported greater discomfort on the side treated with the PRF‐impregnated collagen matrix, while the remaining 12 reported greater discomfort on the side treated with collagen matrix alone, indicating no preference between treatments (Table S1).

### 3.4 Esthetic Outcomes

Based on the secondary outcome of RES, mean scores for the test and control groups were 5.79 ± 2.48 vs. 6.04 ± 3.36, respectively. No significant differences were observed between groups at the 6‐month evaluation (*p* = 0.719; Table S2). Inter‐examiner reliability analysis demonstrated excellent agreement between examiners (ICC = 0.915; 95% CI: 0.854–0.952), indicating a high level of consistency between measurements obtained by the two examiners. The intra‐examiner ICC for the total RES score demonstrated good agreement for Examiner 1 (ICC = 0.842) and excellent agreement for Examiner 2 (ICC = 0.935), indicating a high level of measurement reproducibility.

### 3.5 Data From Digital Models Assessment

In the intragroup comparison (baseline vs. 6 months), the test group showed a statistically significant improvement from baseline to 6 months (*p* = 0.001). A significant increase in root coverage was observed over time, with a mean difference of 0.67 ± 0.04 (median: 0.83; 95% CI: 0.09–1.01). Similarly, the control group demonstrated a statistically significant change between baseline and 6 months (*p* < 0.001), with a mean difference of 0.69 ± 0.01 (median: 0.76; 95% CI: 0.25–1.02). When the intergroup comparison at 6 months was evaluated, no statistically significant difference was observed between the test and control groups (*p* = 0.771), indicating comparable outcomes between the two treatment approaches at the 6‐month follow‐up. More details in Table 3.

**TABLE 3:** Root coverage assessment in digital models at baseline and 6 months for test and control groups.

Patient	Test							Control							Test vs control at 6 months		
	Baseline mean (SD)	6 months mean (SD)	*Difference* mean (SD)	Total mean (SD)				Baseline mean (SD)	6 months mean (SD)	*Difference* mean (SD)	Total mean (SD)				Median	IC 95%	*p* value
1	2.21 (0.69)	1.77 (1.00)	0.44 (−0.31)	*Baseline* 1.93 (0.32)	*Median* *Baseline* vs *6 months*	*IC 95%* *Baseline* vs *6 months*	*p value* *Baseline* vs *6 months*	2.34 (0.68)	2.18 (0.43)	0.16 (0.25)	*Baseline* 1.96 (0.38)	*Median* *Baseline* vs *6 months*	*IC 95%* *Baseline* vs *6 months*	*p value* *Baseline* vs *6 months*	−0.0100	−0.280 to 0.190	0.771
2	1.59 (1.26)	1.83 (0.64)	−0.24 (0.62)		0.830	0.0900 to 1.01	0.001	1.44 (0.96)	1.10 (0.62)	0.34 (0.34)		0.760	0.250 to 1.02	< 0.001			
3	1.23 (0.44)	1.12 (0.45)	0.11 (−0.05)					1.74 (0.41)	1.40 (0.57)	0.34 (−0.17)							
4	1.07 (0.55)	1.12 (0.45)	−0.05 (0.10)					1.48 (1.33)	1.40 (0.65)	0.07 (0.68)							
5	1.49 (0.80)	1.67 (0.79)	−0.17 (0.01)					1.98 (0.96)	1.80 (0.90)	0.19 (0.07)							
6	3.09 (0.50)	1.19 (0.82)	1.90 (−0.32)					3.68 (1.22)	1.60 (0.70)	2.08 (0.52)							
7	1.16 (0.88)	1.07 (0.77)	0.09 (0.11)					0.99 (0.91)	0.74 (0.98)	0.25 (−0.07)							
8	1.55 (0.55)	0.50 (0.86)	1.06 (−031)					1.50 (0.49)	0.42 (0.72)	1.08 (−0.23)							
9	2.34 (0.92)	1.49 (0.56)	0.86 (0.37)	*6 months* 1.26 (0.28)				1.54 (1.04)	0.71 (0.34)	0.83 (0.71)	*6 months* 1.27 (0.36)						
10	2.27 (0.39)	1.54 (0.89)	0.72 (−0.49)					2.06 (0.65)	1.23 (0.52)	0.83 (0.13)							
11	2.38 (0.68)	0.67 (0.10)	1.72 (0.59)					1.87 (0.78)	0.48 (0.07)	1.40 (0.71)							
12	1.38 (0.73)	0.49 (0.27)	0.89 (0.46)					1.47 (0.48)	0.51 (0.33)	0.95 (0.15)							
13	2.00 (0.90)	0.93 (0.27)	1.07 (0.64)					2.11 (0.32)	0.86 (0.89)	1.26 (− 0.57)							
14	2.70 (0.40)	0.81 (0.39)	1.89 (0.01)					1.76 (0.70)	0.90 (0.35)	0.86 (0.35)					Median	IC 95%	*p* value
15	2.33 (1.36)	1.52 (0.63)	0.81 (0.73)					1.60 (0.80)	0.91 (0.15)	0.69 (0.65)					−0.0800	−0.190 to 0.280	0.795
16	1.95 (1.25)	0.94 (0.54)	1.02 (0.72)					1.47 (1.30)	0.84 (023)	0.63 (1.07)							
17	2.16 (1.03)	3.19 (0.79)	−1.04 (0.24)	*Difference* 0.67 (0.04)				4.19 (1.82)	3.65 (1.53)	0.54 (0.29)	*Difference* 0.69 (0.01)						
18	2.07 (0.99)	1.14 (0.66)	0.92 (0.32)					1.91 (0.79)	0.89 (0.46)	1.02 (0.33)							
19	1.04 (0.16)	1.23 (0.18)	0.16 (−0.02)					2.17 (0.98)	1.23 (0.45)	0.94 (0.53)							
20	1.36 (0.15)	1.40 (0.56)	−0.04 (−0.41)					1.35 (0.04)	1.80 (0.91)	−0.45 (−0.87)							
21	2.59 (0.86)	1.66 (1.07)	0.93 (−0.21)					1.69 (1.01)	1.49 (1.03)	0.20 (−0.02)							
22	2.56 (0.73)	1.21 (0.72)	1.35 (0.01)					2.67 (0.97)	1.39 (1.36)	1.28 (−0.39)							
23	1.89 (0.78)	0.94 (0.55)	0.90 (0.23)					1.87 (1.16)	1.94 (0.58)	−0.07 (0.58)							
24	1.62 (0.75)	0.72 (0.07)	0.90 (0.68)					2.28 (0.76)	1.02 (0.42)	1.26 (0.33)							

Abbreviation: SD = standard deviation.

## 4 Discussion

This split‐mouth randomized clinical trial assessed whether PRF impregnation of a volume‐stable collagen matrix would enhance clinical, esthetic, and patient‐reported parameters related to root coverage of multiple gingival recessions employing the coronally advanced flap technique. The results indicated improvements in some clinical parameters, namely RD, CAL, GT, and GP, for both groups, with or without PRF association. These findings suggest the effectiveness of coronally advanced flap combined with collagen matrices as a possible treatment for multiple adjacent recessions, consistent with existing literature supporting the success of coronally advanced flap with soft tissue substitutes [4, 9, 11, 12]. Although both groups exhibited significant intragroup changes from baseline to 6 months in many clinical parameters, no statistically significant intergroup differences were observed at 6 months. The average reduction in RD relative to the baseline was about 1.7 mm for the test group and 2.0 mm for the control group, which aligns with the findings of a case series that examined VCMX + PRF in single‐tooth gingival recessions [38]. The difference from baseline to six‐month evaluation at CAL gain was approximately 1.2 and 1.4 mm for test and control, respectively, showing no relevant differences between groups. These results indicate that PRF impregnation did not enhance root coverage outcomes compared to collagen matrix alone, demonstrating minimal added value of this biologic adjunct in root coverage procedures.

A potentially relevant clinical finding was the better maintenance of the keratinized tissue band in the test group, which showed a mean reduction of approximately 0.5 mm compared to 0.6 mm in the control group. Even though the difference is not clinically relevant, we speculate that the observed variation between PRF‐impregnated and non‐impregnated matrices may result from a potential biological role of PRF in promoting epithelial differentiation or keratinization. The regenerative capacity of PRF is supported by its content of growth factors like TGF‐β and VEGF, which are involved in soft tissue remodeling and angiogenesis [25, 39]. A meta‐analysis reported that, among the various platelet concentrate preparation protocols, PRF was the only modality that significantly enhanced clinical root coverage outcomes compared with CAF alone [40]. This performance has been attributed to the gradual and sustained release pattern of growth factors from PRF, which promotes more efficient inflammatory resolution, accelerates re‐epithelialization, and contributes to more stable tissue closure, thereby reducing soft‐tissue contraction. However, in our study, this isolated improvement in KTW did not translate to superior outcomes in GT or esthetics. Potential explanations include inadequate retention of liquid PRF in the scaffold, suboptimal release dynamics, or saturation limits within the collagen matrix.

The absence of statistically significant intergroup differences in our study aligns with the long‐term observations of trials using other biologics, such as recombinant human platelet‐derived growth factor [41, 42]. Although growth factors may accelerate initial tissue thickening or volumetric scores, they do not appear to alter the fundamental clinical plateau achieved by a volume‐stable matrix, suggesting a “saturation point” in which the mechanical protection of the scaffold provides the necessary environment for healing, rendering the addition of autologous blood concentrates like PRF a negligible adjunct in the presence of a robust collagenous framework. Evidence from clinical studies demonstrated that treating RT1 gingival recessions associated with non‐carious cervical lesions with CAF associated with VSCM significantly enhanced GT gain compared with CAF alone [43]. Nonetheless, no significant differences were observed in recession reduction after 6 months, suggesting that the primary advantage of VSCM may be soft tissue volume augmentation rather than improved root coverage outcomes. In this context, the present clinical trial directly contributes to this discussion by testing a VSCM impregnated with PRF. Despite the biological mechanisms attributed to PRF, our findings suggest that the addition of liquid PRF does not provide relevant short‐term benefits in terms of recession reduction or percentage of root coverage.

At the single‐case level, a previous report documented complete root coverage, increased gingival thickness, and a 1‐mm gain in keratinized mucosa width after treating a maxillary canine recession with a VCMX biofunctionalized with injectable PRF combined with CAF [26]. Those findings supported the hypothesis that PRF‐mediated angiogenesis and growth‐factor release could enhance VCMX performance and facilitate handling. Although our clinical trial confirms the improved intraoperative handling of PRF‐impregnated matrices, this observation is based solely on the surgeon's subjective perception because no methodological assessment of handling was performed. However, the magnitude of soft‐tissue gains described in that previous report was not replicated in our findings. In particular, the modest between‐group difference in KTW reduction (−0.5 vs. −0.6 mm) and the absence of superiority in GT or %CRC suggest that the favorable outcomes previously reported may be patient‐ or defect‐specific, highlighting the need for well‐controlled clinical trials to validate such approaches.

Regardless of PRF impregnation, an improvement in the esthetics was observed through RES in both groups treated with collagen matrices. The absence of statistically significant differences between test and control groups suggests that PRF does not provide additional esthetic benefits beyond those achieved with the collagen matrix alone. Additionally, patient‐reported outcome measures (PROMs) indicated no differences between groups in terms of discomfort, pain, swelling, or recovery experiences, reinforcing the marginal benefits of PRF addition on VSCM. When patients were asked which side they perceived as more uncomfortable postoperatively, only half of them reported less discomfort with the PRF impregnation. In other studies, comparisons made between coronally advanced flap associated with connective tissue graft (CTG + CAF) and CAF + VSCM showed that patients reported a preference for the CAF + VSCM technique [35].

Considering the clinical relevance of our results, the differences observed in clinical measurements did not consistently favor the PRF functionalization of collagen matrices applied in root coverage procedures. The marginal gain in KTW, without accompanying benefits in other clinical, esthetic, or patient‐reported outcomes, indicates a limited clinical impact for using PRF in this specific setting. Although PRF has shown promising outcomes in regenerative medicine [44, 45], our results evidenced that its effectiveness is procedure and context‐dependent. Despite this, it was evident that the addition of PRF favors the manipulation of the collagen matrix and its placement in the surgical site. The impregnation of liquid PRF into the collagen matrix avoided ripping during matrix suture, facilitating the procedure and potentially reducing surgical time. This clinical perception is consistent with previous observations [27, 38], who also noted improved handling characteristics when collagen matrices were biofunctionalized with liquid PRF. This aspect should be considered by clinicians when deciding whether to use PRF‐functionalized collagen matrices in clinical practice.

It is noteworthy that CAF alone is a predictable and effective treatment for RT1 gingival recessions, particularly in maxillary sites with favorable anatomy, including adequate keratinized tissue, gingival thickness, and well‐preserved interdental papilla [30]. Such conditions facilitate stable flap positioning and may limit the incremental benefits that biologics or biomaterials can provide, especially in short‐term evaluations [39]. Histological evidence also indicates that healing in these scenarios occurs predominantly through long junctional epithelium and connective tissue adhesion, which is generally sufficient to ensure stability in RT1 defects [46]. Within this context and considering that our trial compared two matrix‐based approaches, CAF alone would likely have yielded satisfactory outcomes in some cases, making the clinical scenario insufficiently demanding to reveal subtle advantages associated with PRF impregnation.

Limitations of this study include the short‐term results of a 6‐month follow‐up evaluation. Long‐term outcomes could assist the scientific community in clarifying whether PRF offers sustained or delayed benefits in soft tissue augmentation. Moreover, further long‐term clinical investigations are needed to fully elucidate the role of PRF as an instrument for biofunctionalization of xenogeneic scaffolds. In addition, future trials should assess differences in PRF presentation, concentration, and retention methods to optimize its integration into surrounding tissues. We also recommend further preclinical investigations to explore the histological aspects of root coverage with collagen matrices functionalized with PRF. Although antibiotics were prescribed to patients following surgery in this clinical trial, it is important to emphasize that antibiotic prescriptions should not be made solely to ensure consistency among studies. Considering that gingival recession defects represent some of the cleanest sites within the oral cavity, the risk of infection after soft tissue manipulation in these areas is minimal. Furthermore, the current literature has not yet established clear evidence supporting the routine use of antibiotics for these surgical procedures. Other clinical protocols adopted in this trial, such as the matrix stabilization approach, the trimming of matrices, and the chlorhexidine protocol initiated on the third postoperative day represent methodological choices that, although clinically motivated, were not independently validated, and their potential influence on the outcomes should be considered when interpreting the results. Another aspect for consideration was the statistical power. Although this represents a methodological limitation and warrants cautious interpretation of potential Type II error, our findings still provide valuable insights given the rigor of the clinical protocol, the randomized design, and the consistency of observed outcomes across both treatment arms. Moreover, other studies in the literature showed a high SD when using xenogeneic matrices for soft tissue reconstruction [47], which may be the key point that impacted our analysis. Future studies with larger samples are warranted to confirm and expand upon these findings.

The authors also acknowledge that exploring differences between cases with lower and higher root coverage outcomes (e.g., lowest vs. highest quartiles) could provide valuable insights into potential prognostic factors. However, such analyses were beyond the scope and statistical power of the present study, and warrant investigation in specifically designed trials. An important consideration is that, while GT and GP are related parameters, they reflect different biological characteristics. GT provides a continuous quantitative measurement, whereas GP is a categorical assessment based on probe transparency. Because the transparency threshold lies close to 1.0 mm, small increases in GT may be sufficient to shift a site from a thin to a thick phenotype, even if still below the numerical cutoff of 1.0 mm. This likely explains the higher percentage of thick phenotypes observed at 6 months, despite GT values below 1.0 mm in some cases. For the digital measurements, although performed by an independent and trained examiner, no formal calibration or intra‐examiner reliability assessment was conducted, which should be addressed in future studies.

Another limitation concerns esthetic assessment. Esthetics was evaluated through the RES by calibrated examiners, which may not fully reflect the patient‐centered esthetic impact. Although PROMs captured postoperative symptoms, they did not include patients' perception of esthetic improvement. Future studies should incorporate validated esthetic PROMs to complement examiner‐based evaluations. Furthermore, dentin hypersensitivity was not evaluated using clinical or validated patient‐reported instruments in this trial. Future investigations should include standardized assessments of hypersensitivity to provide a more comprehensive evaluation of functional patient outcomes. Blinding of the clinical examiner was not performed in this trial. The primary outcome examiner also performed the surgical procedures in the majority of cases (22 of 25), creating a dual role that represents a relevant risk of detection bias. The standardized measurement protocol and the split‐mouth randomized design could partially mitigate this limitation, but the potential influence of the examiner awareness of treatment allocation on outcome assessment cannot be entirely excluded. We recommend future trials to prioritize the use of independent examiners with no involvement in the intervention phase. Another methodological limitation concerns the involvement of two non‐calibrated operators in the surgical procedures. Although both surgeons were experienced and followed a predefined standardized protocol, this represents a methodological limitation, as the absence of a structured calibration process limits the ability to objectively verify inter‐operator consistency. Adherence to the standardized protocol was maintained throughout the trial. However, some degree of operator‐related variability cannot be entirely excluded and may have introduced a source of performance bias. Future studies should incorporate formal calibration procedures or, where feasible, restrict procedures to a single operator. Future studies conducted by a single surgeon or incorporating operator effects into the statistical analysis may minimize this potential source of bias.

## 5 Conclusions

Using a volume‐stable collagen matrix biofunctionalized with liquid PRF did not provide additional benefits in recession depth reduction compared with the collagen matrix alone after 6 months. Both approaches promoted comparable clinical improvements in RT1 multiple recessions, and therefore PRF impregnation did not enhance short‐term root coverage outcomes in this setting. Improved handling and suture capacity were noticed by surgeons with PRF‐impregnated matrices. However, these potential technical advantages were not objectively assessed and therefore cannot be conclusively supported by the present study. The comparison of two collagen matrix–based approaches was conducted in a relatively straightforward clinical scenario (RT1 maxillary defects), which may have limited the ability to detect subtle or additive effects of PRF beyond those already achievable with CAF‐based therapy. Future research should explore more demanding cases or include a CAF‐only control to clarify potential benefits of PRF.

## Funding

This work was supported by Osteology Foundation, 20127.

## Disclosure

AI statement: Artificial intelligence tools (ChatGPT 4.0) were used solely for language editing and proofreading (grammar and spelling) of an entirely human‐generated manuscript. No AI system was used for data analysis, interpretation, figure generation, or content creation.

## Conflicts of Interest

The authors declare no conflicts of interest. The authors do not have any financial interest in companies whose materials are included in this article.

## Supporting information


**Figure S1:** Clinical measurements. (a) Recession depth; (b) Width of the keratinized tissue band; (c) Probing depth at the mid‐buccal site; (d, e) Gingival thickness.
**Figure S2:** Graphs of paired analyses within the test group comparing baseline (T0) with the 6‐month follow‐up (T1).
**Figure S3:** Graphs of paired analyses within the control group comparing baseline (T0) with the 6‐month follow‐up (T1).
**Figure S4:** Graphs of paired data analyses between the test and control groups at the 6‐month follow‐up (T1).
**Table S1:** Cross‐tab with visual analogue scale (VAS) values obtained from the questionnaires answered by the patients (PROMs).
**Table S2:** Root Coverage Esthetic Score (RES) evaluation.

## Data Availability

The data that support the findings of this study are available from the corresponding author upon reasonable request.
