# Supplementary material for: Volume‐Stable Collagen Matrix With or Without Platelet‐Rich Fibrin Impregnation for Multiple Gingival Recessions: A Randomized Clinical Trial
Source: J Periodontal Res. 2026 Jun 25;61(8):929–43. doi: 10.1111/jre.70127 (PMC13703892; doi:10.1111/jre.70127)
Supplement: Supplementary file 1 [file JRE-61-929-s001.docx]

**Supplementary File**

**Supplementary Figure 1.** Clinical measurements.

**Supplementary Figure 1.** **Clinical measurements. (a) Recession depth; (b) Width of the keratinized tissue band; (c) Probing depth at the mid-buccal site; (d-e) Gingival thickness.**

**Supplementary Figure 2.** Graphs of paired analyses within the test group comparing baseline (T0) with the 6-month follow-up (T1).

**Legend:**(a) Recession Depth (RD), (b) Keratinized Tissue Width (KTW), (c) Probing Depth (PD), (d) Gingival Thickness (GT), (e) Clinical Attachment Level (CAL).

**Supplementary Figure 3.** Graphs of paired analyses within the control group comparing baseline (T0) with the 6-month follow-up (T1).

**Legend:** (a) Recession Depth (RD), (b) Keratinized Tissue Width (KTW), (c) Probing Depth (PD), (d) Gingival Thickness (GT), (e) Clinical Attachment Level (CAL).

**Supplementary Figure 4.** Graphs of paired data analyses between the test and control groups at the 6-month follow-up (T1).

**Legend: (a) Recession Depth (RD), (b) Keratinized Tissue Width (KTW), (c) Probing Depth (PD), (d) Gingival Thickness (GT), (e) Clinical Attachment Level (CAL).** (PD), (d) Gingival Thickness (GT), (e) Clinical Attachment Level (CAL).

**Supplementary Table 1.** Cross‐tab with visual analogue scale (VAS) values obtained from the questionnaires answered by the patients (PROMs).

|  | **Test** | **Control** | **P-value** |
| --- | --- | --- | --- |
| **Q1 Mean (SD)** | 4.13 (2.47) | 3.50 (2.50) | .412 |
| **Q2 Mean (SD)** | 2.57 (2.42) | 3.00 (2.79) | .321 |
| **Q3 Mean (SD)** | 2.52 (2.56) | 3.00 (3.17) | .280 |
| **Q4 Mean (SD)** | 1.60 (2.27) | 1.79 (2.17) | .390 |
| **Q5 Mean (SD)** | 3.68 (2.79) | 4.92 (3.12) | .165 |
| **Q6 Mean (SD)** | 1.66 (2.39) | 2.18 (3.11) | .361 |
| **Q7 Mean (SD)** | 41.67 (50.36) | 58.33 (50.36) | .541 |

**Q1:** How was the discomfort during the surgery for root coverage on the right/left side? **Q2:** Did you feel pain during the surgery for covering the dental roots on the right/left side? **Q3**: Did you feel pain after the surgery for covering the dental roots on the right/left side? **Q4:** Was there post-operative bleeding on the right/left side? **Q5:** How would you rate the swelling on the right/left side? **Q6:** Did you notice any color change (bruising) on the right/left side of your face? **Q7:** Overall, which side caused you to feel more discomfort (less than 50% represent test side and more than 50% represent control side)?

**Supplementary Table 2.** Root Coverage Esthetic Score (RES) evaluation.

| **Groups** | **Mean (SD)** | **Median (Q1-Q3)** | ***P value*** *(Test vs Control)* |
| --- | --- | --- | --- |
| **Test** | 5.79 (2.48) | 7.00 (5.25-7.00) | *p=.719* |
| **Control** | 6.04 (3.36) | 6.50 (4.25-9.5) |  |

Legend: SD=standard deviation; Q1=25% percentile; Q3=75% percentile.
